# An Improved Method for Determining the Infection Titer of Replication-Competent Adeno-Associated Virus

**DOI:** 10.3390/biomedicines14030653

**Published:** 2026-03-13

**Authors:** Jianning Fu, Lei Yu, Zhihao Fu, Guangyu Wang, Chenggang Liang, Xinchang Shi, Yixuan Zhang

**Affiliations:** 1 School of Life Science and Biopharmaceutics, Shenyang Pharmaceutical University, No. 103 Wenhua Road, Shenyang 110016, China; 2 Key Laboratory of the Ministry of Health for Research on Quality and Standardization of Biotech Products, National Institutes for Food and Drug Control, No. 2, Tiantan Xili, Dongcheng District, Beijing 100050, China

**Keywords:** replication-competent adeno-associated virus, infectious titer, TCID_50_ assay

## Abstract

**Background/Objectives**: Recombinant adeno-associated virus (rAAV) has become a leading vector in gene therapy. However, manufacturing limitations may result in replication-competent AAV (rcAAV) contamination of clinical rAAV products, posing safety risks. Rigorous testing is therefore essential, and the use of accurately calibrated rcAAV reference standard materials is critical for ensuring assay stability and reliability. A disadvantage of the widely used Tissue Culture Infectious Dose 50 (TCID_50_) assay is its high variability. This study introduces an optimized TCID_50_ assay for the precise quantification of infectious rcAAV particles. **Methods**: We developed a TCID_50_ assay tailored to *rep2*-based rcAAV, optimizing key aspects such as viral infection conditions, qPCR reaction systems, and standard curve preparation. We employed an innovative strategy to prepare the standard curve using serial dilutions of rcAAV in cell lysate, ensuring alignment with the test sample matrices. **Results**: The rcAAV-derived standard curve demonstrated exceptional linearity (R^2^ > 0.99), sensitivity (LOQ ≈ 38 copies), and reproducibility, enabling robust endpoint qPCR analysis. The optimized assay significantly improved the precision of the TCID_50_ assay, as an inter-assay coefficient of variation (CV) of 11.4% was achieved. **Conclusions**: This refined TCID_50_ assay is a reliable method for calibrating infectious titers of rcAAV reference standard materials, thereby enabling the standardization of rcAAV testing.

## 1. Introduction

Recombinant adeno-associated virus (rAAV) has emerged as a leading tool in gene therapy due to its favorable safety profile, broad tropism, and capacity for sustained transgene expression [1,2,3]. The growing number of approved AAV-based therapies and an expanding clinical pipeline underscore its significant therapeutic potential [2,4,5]. Ensuring the safety and efficacy of clinical treatments relies heavily on comprehensive quality analysis and risk monitoring of rAAV products using multiple analytical methods [6,7,8]. Currently, the industrial-scale production of rAAV primarily relies on two systems: HEK293 cell-based transient transfection and the baculovirus/Sf9 insect cell system [9,10]. A fundamental and universal characteristic of these platforms is the introduction of essential AAV *rep* and *cap* genes into producer cells to facilitate viral replication and capsid formation. However, the use of this design inherently risks the generation of replication-competent AAV (rcAAV) through recombination between vector genomes (flanked by inverted terminal repeats, ITRs) and the supplied *rep/cap* sequences [11,12,13,14]. The presence of rcAAV in clinical batches poses substantial long-term safety concerns, including immunotoxicity and carcinogenicity, driven by persistent expression of Rep and Cap proteins. Although there are many processes that have undergone improvements to significantly reduce the production of rcAAV, they have not yet been adopted for use in clinical batches [15,16]. Regulatory agencies now mandate the rigorous detection of rcAAV contamination in clinical AAV products.

Given that residual plasmid DNA (in three-plasmid systems) or baculoviral genomic fragments (in Sf9 systems) may cause false positives in quantitative polymerase chain reactions (qPCRs), current rcAAV detection strategies employ multi-round cell passage coupled with qPCR [13,14]. This approach enriches rcAAV while diluting background nucleic acids, thereby enhancing sensitivity and specificity. The assay requires the inclusion of a well-qualified and homogeneous rcAAV reference as a limit test control, and test validity is contingent upon detectable amplification of this control. The titer of the limit test control is essential for degerming rcAAV levels in test samples [14]. For instance, with a limit test control of 10 infectious units and a test sample load of 1 × 10^10^ vector genomes (vg), the result is reported as <10 infectious units per 1 × 10^10^ vg rAAV if the test sample shows no amplification, while the control amplifies as expected. Therefore, the reliability of this method depends critically on the availability of accurately calibrated rcAAV reference standard materials. It should be noted that while rcAAV levels are sometimes reported in vector genome (vg) units, rcAAV testing specifically detects active AAV particles that are capable of causing infection and replicating. For AAV viruses, the genome titer may be overestimated due to the presence of unpackaged nucleic acids that are not encapsulated. Moreover, even if the genome is successfully packaged within the capsid, the virus may still lack infectivity—either because the capsid itself fails to mediate cell entry or because the packaged genome is incomplete and unable to replicate upon cell entry. Such particles do not constitute an infectious virus. So, a significant and well-recognized discrepancy exists between the genomic titer and the infectious titer of AAV particles [7,8]. Infectious activity can vary considerably across different serotypes and even different production processes [9,17]. Consequently, it is not appropriate to report rcAAV testing results in vg units; instead, we strongly recommend that rcAAV reference standard materials be calibrated in infectious titers.

The infectious titer of AAV is currently determined using the Tissue Culture Infectious Dose 50 (TCID_50_) assay, which remains the gold standard for quantifying viral infectivity [18]. However, as AAV does not produce cytopathic effects, quantitative PCR (qPCR) is employed to identify infected wells [19]. This approach has inherent limitations affecting its accuracy and precision. The high coefficient of variation (CV) primarily stems from factors including cell culture variability, helper virus instability, operational inconsistencies, and interference from lysis buffer components (e.g., PCR inhibition). The rAAV reference materials from the American Type Culture Collection (ATCC) are typically calibrated for infectious titer using the TCID_50_ assay. However, the disclosed calibration data reveal substantial variability. Taking the rAAV2 reference standard material (VR-1616) as an example, the mean calibrated infectious titer is 7 × 10^9^ IU/mL, with a standard deviation (SD) of 6.7 × 10^9^ IU/mL, resulting in a CV as high as 95.7% [19]. Similarly, for the rAAV8 reference standard material (VR-1816), the mean infectious titer is 2.67 × 10^9^ IU/mL, with an SD of 3.32 × 10^9^ IU/mL and a CV reaching 124% [20]. These data indicate that the variability in the test results obtained using the current TCID_50_ method is substantial. Despite the considerable optimization efforts that have been exerted in recent years, the CV consistently exceeds 70% [21,22,23,24].

To address this critical limitation, we developed an optimized TCID_50_-based assay to quantify infectious particles in rcAAV reference standard materials. Our method specifically addresses the shortcomings of existing protocols by providing improvements in viral infection parameters, qPCR reaction conditions, and standard curve preparation. We demonstrate the application of this refined protocol to accurately determine the infectious titer of a *rep2*-based recombinant rcAAV8 sample.

## 2. Materials and Methods

### 2.1. Materials

Type 5 adenovirus (Ad5) and HEK293 and HEK293T cells were obtained from the American Type Culture Collection (ATCC, Manassas, VA, USA). Ad5 was amplified in HEK293 cells, and the resulting purified virus stock was qualified, and its infectious titer was determined to be 1.71 × 10^10^ TCID_50_/mL by TCID_50_ assay. Type 8 rcAAV (rcAAV8) was generated through a two-step process: initial production of virus seeds by co-transfecting HEK293 cells with a *rep2-cap8* plasmid (containing ITRs) and a helper plasmid, followed by amplification using Ad5 helper virus. The harvested rcAAV8 was purified using ultracentrifugation, affinity chromatography, and ion-exchange chromatography. PackGene Biotechnology (Guangzhou, China) was used for the preparation of the rcAAV8 sample. Probe-based qPCR master mixes (cataloged as A–F) were sourced from the following manufacturers: Roche (Basel, Switzerland), Applied Biosystems (Foster City, CA, USA), Takara Bio (Shiga, Japan), Tsingke Biotechnology (Beijing, China), Vazyme Biotech (Nanjing, Jiangsu, China), and Yeasen Biotechnology (Shanghai, China).

### 2.2. Primers and Probes

The primers and fluorescent probes used to target the *cap8*, *rep2*, and ITR regions were custom-synthesized by Sangon Biotech (Shanghai, China). Sequence details are provided in Table 1.

### 2.3. Digital PCR for Genomic Titer Determination

The rcAAV8 sample was mixed with lysis buffer (62.5 mM EDTA·2Na, 0.1% SDS, pH 8.0) in a 1:1 ratio and incubated at 95 °C for 10 min, followed by rapid cooling to 4 °C. The mixture was diluted to the appropriate concentration using TE buffer supplemented with 0.05% F-68 before undergoing digital PCR analysis.

Digital PCR was performed on the Bio-Rad QX200™ Droplet Digital™ PCR system equipped with the AutoDG™ Automated Droplet Generator (Bio-Rad, Hercules, CA, USA). Each 20 µL reaction contained 10 µL of ddPCR Supermix for Probes (No dUTP), 5 µL of template DNA, 1.8 µL each of forward and reverse primers (10 µM), 0.5 µL of probe (10 µM), and 0.9 µL of nuclease-free H_2_O. The mixture was loaded into a DG8™ Cartridge, and droplets were generated automatically using the AutoDG™ instrument (Bio-Rad, Hercules, CA, USA) with 70 µL of Droplet Generation Oil per sample, yielding ~20,000 droplets per reaction. Thermal cycling conditions included an initial denaturation step at 96 °C for 10 min, followed by 40 cycles of denaturation at 95 °C for 30 s, annealing at 60 °C for 30 s, and extension at 72 °C for 15 s, concluding with a final denaturation step at 98 °C for 10 min. Post-PCR droplets were analyzed using the QX200™ Droplet Reader (Bio-Rad, Hercules, CA, USA), and absolute quantification was determined using QuantaSoft software v1.7.4, where the Poisson algorithm was employed.

### 2.4. TCID_50_ Procedure

#### 2.4.1. Sample Preparation

RcAAV8 samples were serially diluted 10-fold in Opti-MEM supplemented with Ad5 to achieve final dilution factors ranging from 10^−5^ to 10^−10^. HEK293T cells in the logarithmic growth phase were harvested and adjusted to a density of 1.8 × 10^6^ cells/mL. A total of 50 μL of each rcAAV8 dilution (10^−5^–10^−10^) was combined with 50 μL of cell suspension in 96-well plates, with twelve replicates per dilution distributed across rows A–F. Rows G–H received 50 μL of Opti-MEM supplemented with Ad5 (MOI = 5.5) and 50 μL of cell suspension. The plate was sealed with a breathable membrane and incubated at 37 °C for 72 h; during this period, the rcAAV sample at a 10^−1^ dilution (designated RS1) was archived at −20 °C for later use. Following incubation, 50 μL of supernatant was removed from wells G1–G8 and H1–H8. The RS1 stock underwent additional 10-fold serial dilutions to generate eight standards (RS1–RS8; 10^−1^ to 10^−8^). Each standard (50 μL) was added in duplicate to wells G1–G8 and H1–H8 to generate a standard curve, while the remaining eight wells (G9–G12 and H9–H12) served as the negative controls. All wells then received 90 μL of freshly prepared lysis buffer (0.5% sodium deoxycholate, 0.9% Tween-20, 0.6 mg/mL proteinase K in 1× proteinase K buffer), sealed with PCR adhesive film, and they were subjected to a stepwise lysis protocol: 37 °C for 1 h, 55 °C for 2 h, 95 °C for 30 min, followed by storage at 4 °C until they underwent qPCR analysis (completed within one week).

#### 2.4.2. qPCR Detection

The qPCR reaction mixtures were prepared in a total volume of 27.5 μL per well, containing 15 μL of 2× qPCR TaqMan Probe Master Mix, 1 μL each of forward (F) and reverse (R) primers (10 μM), 0.5 μL of TaqMan probe (10 μM), 0.6 μL of ROX reference dye (50×), and 9.4 μL of nuclease-free H_2_O. After thorough vortexing and brief centrifugation, 27.5 μL of the pre-mixed reaction solution was dispensed into each well of a 96-well PCR plate. Subsequently, 2.5 μL of cell lysate was added to each well, followed by brief centrifugation (300× *g* for 1 min) to collect all liquid at the bottom of the wells. The qPCR amplification was performed using a QuantStudio™ 7 Flex Real-Time PCR System (Thermo Fisher Scientific, Waltham, MA, USA) with the following thermal cycling conditions: initial denaturation at 95 °C for 10 min, followed by 40 cycles of denaturation at 95 °C for 15 s and combined annealing/extension at 60 °C for 60 s. Fluorescence signals were acquired during the annealing/extension phase.

#### 2.4.3. Positive Judgment and TCID_50_ Calculation

A standard curve was generated via linear regression of log_10_-transformed genomic copy numbers (RS1–RS7) against their corresponding CT values. This curve was then used to calculate genome titers for all test wells in rows A–F. Adjusted titers (increased titers) were computed by subtracting both the input genome titer and three times the LOQ (38 copies × 3 = 114 copies). Wells with positive adjusted titers were classified as positive, while those with negative values were deemed negative. Infectious titers were subsequently calculated using the Spearman–Kärber formula, detailed as follows:

log_10_ TCID_50_ = −[x_0_ + (d/2) − dΣpᵢ],

where x_0_ is the log_10_ of the highest dilution tested, d is the log_10_ of the dilution factor, and pᵢ is the proportion of positive wells at each dilution.

### 2.5. Generation of Standard Curve Groups

To optimize standard curve preparation, four experimental groups were designed and executed, as detailed in Table 2. In Group 1, serial dilutions of rcAAV8 were co-seeded with HEK293T cells (without Ad5) on day 1. For Groups 2–4, varying combinations of cells and Ad5 were seeded on day 1, with rcAAV8 serial dilutions added immediately prior to the cell lysis on day 3. This approach enabled us to perform a systematic evaluation of cellular and Ad5 contributions to rcAAV genome amplification during qPCR.

## 3. Results

### 3.1. Determination of Genomic Titer of rcAAV8 Using dPCR

**Figure 1:**
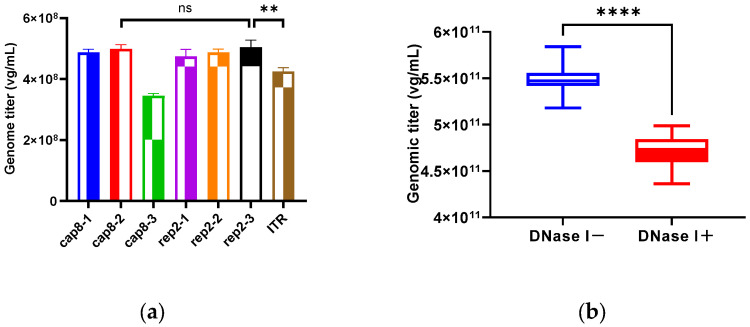
Genomic titer determination of rcAAV8 using digital PCR: (**a**) Primer–probe screening and validation. (**b**) Estimated genomic titers of rcAAV8. “DNase I−” denotes samples without DNase I treatment, while “DNase I+” indicates samples treated with DNase I. Each data point represents the mean of fifteen replicates. “ns” indicates “no significant difference between two groups”, and asterisks indicate the level of significance: ** *p* < 0.01, **** *p* < 0.0001 (*t*-test).

To support the development of the TCID_50_ assay, a recombinant rcAAV8 was produced using a two-plasmid co-transfection system, and a digital PCR method was established for the quantification of the genome titer. Since precise AAV genome quantification depends on rigorously validated primers and probes, we designed and evaluated seven primer–probe sets—three targeting the *cap8* gene, three targeting the *rep2* gene, and one targeting the ITR region. As shown in Figure 1a, the *rep2*-targeting set (*rep2*-3) and the *cap8*-targeting set (*cap8*-2) exhibited the highest amplification efficiency among all the combinations tested. No significant difference was observed between them (*t*-test, *p* = 0.7632), indicating that both are viable candidates. Given its broader applicability across different AAV serotypes, the *rep2*-targeting set was selected for use in further work. This optimized set was then applied to measure the rcAAV8 genomic titer. To differentiate between total and encapsulated genome titers, the assays were performed under two conditions—namely, in the presence or absence of DNase I treatment, which is commonly used to remove unpackaged nucleic acids. As summarized in Figure 1b, the mean titer was 5.47 × 10^11^ vg/mL without DNase I treatment and 4.72 × 10^11^ vg/mL with DNase I treatment. A significant difference was observed between the two groups (*p* < 0.0001, *t*-test), indicating the presence of free genomic DNA in the sample. Given that free genomes may also be amplified during the qPCR analysis, the total genome titer was used for the subsequent TCID_50_ analysis.

### 3.2. Establishment of an Endpoint qPCR for rcAAV Genome Detection in TCID_50_ Assay

The conventional TCID_50_ assay for AAV involves a multi-step process that includes viral infection, intracellular replication, and endpoint qPCR analysis. Endpoint qPCR plays a critical role in identifying positive wells and determining TCID_50_ values. Here, we developed a *rep2* gene-targeted endpoint qPCR assay specifically optimized for the accurate and sensitive quantification of rcAAV genomes in cell lysates. The optimal primer–probe set was validated previously (Section 3.1). This section focuses on optimizing the reaction system to minimize matrix interference caused by cellular components and lysis buffer.

**Figure 2:**
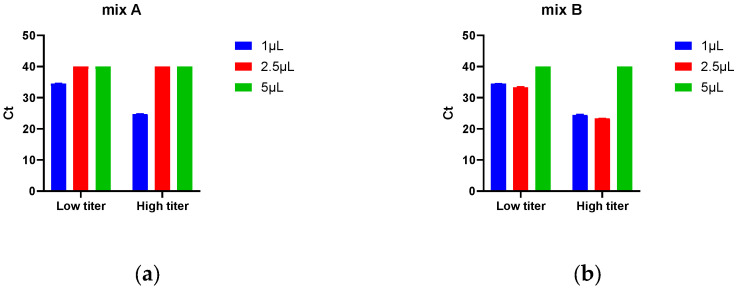

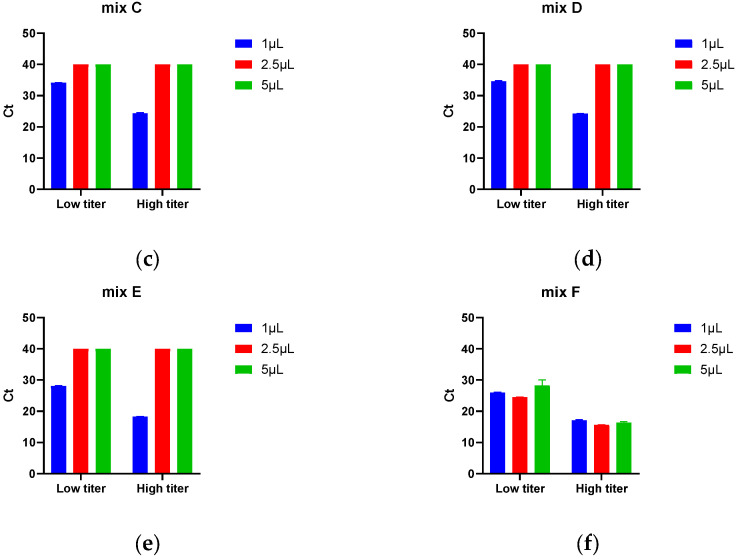
Optimization of endpoint qPCR reaction system: (**a**) Mix A; (**b**) Mix B; (**c**) Mix C; (**d**) Mix D; (**e**) Mix E; (**f**) Mix F. Low titer and high titer represent cell lysates containing low and high copies of rcAAV genomes, respectively. The template volumes tested were 1 μL, 2.5 μL, and 5 μL. Each data point represents the mean of two replicates.

To address the potential inhibition from cell lysates, we tested different volumes of lysate templates containing either low or high concentrations of rcAAV genomes, along with six commercially available qPCR master mixes. As shown in Figure 2, when 5 μL of lysate was used, only Mix F showed detectable amplification. With 2.5 μL of lysate, amplification was observed only with Mixes F and B. At 1 μL of lysate, all mixes supported amplification, but Mix F demonstrated the strongest signal. Overall, Mix F from Yeasen Biotechnology exhibited superior resistance to PCR inhibitors in the lysate matrix. Since mild inhibitory effects were still present with a 5 μL lysate volume, we selected Mix F combined with 2.5 μL of cell lysate for subsequent assays.

### 3.3. Optimization of Virus Infection and Replication Process

In the TCID_50_ assay, the efficiency of viral infection and replication is a critical determinant of the final titer. This section describes the systematic optimization of three key parameters: initial cell seeding density, timing of rcAAV infection, and infection duration.

**Figure 3:**
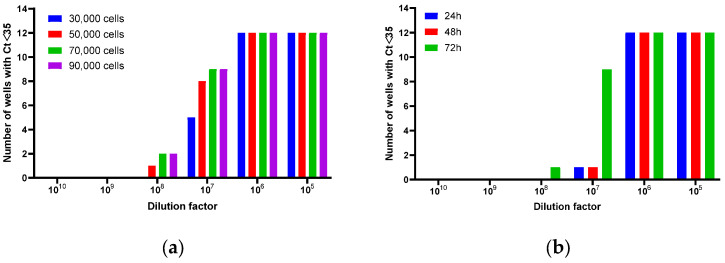
Effect of cell seeding density and infection duration on rcAAV8 replication: (**a**) The number of infected wells (Ct < 35) at different cell seeding densities (30,000 to 90,000 viable cells per well). (**b**) The number of infected wells (Ct < 35) after different infection durations (24 to 72 h) at a seeding density of 90,000 viable cells per well.

As shown in Figure 3a, increasing the cell seeding density from 30,000 to 70,000 viable cells per well enhanced detection sensitivity, as reflected by the rise in wells with a Ct value below 35. The results plateaued between 70,000 and 90,000 cells, suggesting that the positive effect on viral entry and replication reaches an optimum at around 70,000 cells per well. To ensure a robust and conservative experimental condition in subsequent assays, a density of 90,000 cells per well was selected. A similar positive effect was observed when the infection duration was extended, which also led to an increase in the number of wells with Ct < 35 (Figure 3b). Furthermore, concurrent inoculation of cells and rcAAV not only streamlined the experimental workflow but also improved result reproducibility, without significantly altering the final TCID_50_ titer.

Based on these observations, the finalized infection protocol was established as follows: co-inoculation of 90,000 cells per well with rcAAV samples, followed by a 72 h incubation period. Cell lysis was then performed using a previously described method (Section 2.4.1).

### 3.4. Establishment and Evaluation of Standard Curves

Four sets of standard curves were prepared using serially diluted rcAAV8, with specific preparation protocols detailed in Table 2. The input genomic copy number for the RS1 standard was calculated as 5.47 × 10^10^ vg/mL × 0.05 mL = 2.74 × 10^9^ vg. Given a total cell lysate volume of 180 μL (composed of 50 μL of cell suspension, 50 μL of rcAAV dilution, and 90 μL of lysis buffer, with a 10 μL allowance for evaporation during the duration) and a qPCR input volume of 2.5 μL, the theoretical genomic copy number of RS1 in each qPCR reaction was derived as (2.74 × 10^9^ vg/180 μL) × 2.5 μL = 3.8 × 10^7^ vg. Standard curves were generated by performing a linear regression of log_10_-transformed theoretical copy numbers (RS1–RS7) against experimentally measured Ct values.

**Figure 4:**
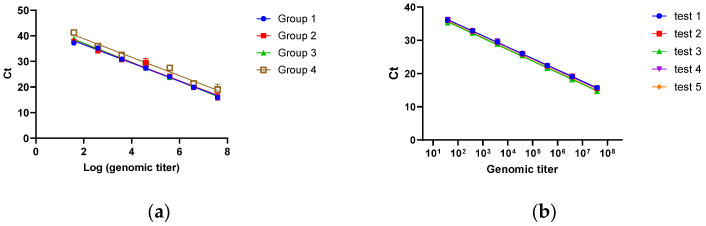
Performance of standard curves prepared from rcAAV8 serial dilutions: (**a**) Standard curves from four preparation groups. (**b**) Repeatability of the third standard curve set across experimental replicates.

As illustrated in Figure 4a, all four standard curves demonstrated satisfactory linearity, with the first three sets showing nearly overlapping regression lines. To ensure matrix consistency between standard and test samples, the third preparation method was selected for subsequent assays. Evaluation of the third standard curve set (Figure 4b, Table 3) confirmed excellent performance, with R^2^ values consistently exceeding 0.99 and amplification efficiencies ranging from 90% to 100% across experimental replicates. The CVs for RS6 and RS7 were 11.5% and 10.6%, respectively. For uninfected control samples, no PCR amplification was detected. Based on this precision profile, the LOQ of the qPCR assay was established at 38 vg, which is comparable to the plasmid-based standard curves reported in previous studies [18,22].

### 3.5. Repeatability of the Optimized TCID_50_ Assay

To assess the repeatability of the developed assay, the rcAAV8 sample underwent five rounds of testing, with three replicates performed in each round. The results are presented in Table 4. Notably, the intra-assay variability remained below 40%, while the inter-assay variability was recorded at 11.4%, which represents a significant improvement over the best-reported inter-assay variability in the literature of >30% [24].

## 4. Discussion

Since rcAAV generated during rAAV production inherently contains the *rep* (predominantly *rep2*) from the packaging plasmid, rcAAV reference materials produced via a two-plasmid system—which mimics this natural process—are more suitable as positive controls for rcAAV testing than wild-type AAV. Accurate determination of the infectious titer of rcAAV reference standards is critical for reliable rcAAV detection. This study established an optimized TCID_50_ method for precise rcAAV infectious titer quantification by systematically refining the key procedural steps, including the viral infection process, qPCR reaction system, and standard curve preparation.

First, the qPCR reaction system was optimized, as the PCR results serve as the primary readout for identifying positive wells. To maximize the positive detection rate, this step requires careful validation. Our investigation yielded two important insights: (1) Cell lysate components substantially inhibit qPCR amplification, necessitating optimization of template volume. Insufficient volumes increase pipetting errors, whereas excessive volumes exacerbate PCR inhibition. (2) Different commercial qPCR master mixes exhibit varying degrees of tolerance to cell lysates. This inhibition likely originates from detergents in the lysis buffer and certain components in cell cultures, while the performance differences among mixes can be attributed to undisclosed variations in DNA polymerase and buffer formulations. Given the difficulty of mechanistically pinpointing the optimal choice without proprietary information, empirical screening proved to be a practical and necessary strategy for selecting the most suitable master mix for endpoint qPCR detection in this system.

Second, the standard curve is fundamental to qPCR quantification and LOQ determination. Although the TCID_50_ assay is well-established, current methods predominantly rely on plasmid DNA as the standard material [18,22], which has notable limitations: (1) plasmid DNA exhibits different amplification efficiency compared with viral genomes, and (2) the matrix effects differ significantly between purified plasmid preparations and complex cell lysates. As demonstrated here, lysis buffer components markedly affect PCR efficiency. A key innovation of our method is the use of the test virus itself to generate standard curves. Specifically, the virus is added to Ad5-infected cells, followed by lysis buffer, exactly mirroring the sample processing steps used in TCID_50_ test wells, thereby ensuring identical sample matrices. To assess potential interference from the cells and Ad5, we designed several standard curve groups. Group 1 (rcAAV8 inoculated into cell plates on day 1 and cultured for three days without Ad5, preventing rcAAV genome replication) did not show lower qPCR amplification than Groups 2 and 3. This indicates that there was no significant loss of rcAAV8 genomes, confirming that non-replicated input rcAAV genomes persist in the lysate and must be deducted in the final qPCR calculations. Groups 2–4 received rcAAV8 on day 3 before cell lysis, with different conditions used to evaluate the effects of cells and Ad5 on rcAAV genome detection: Group 4 contained neither cells nor Ad5, Group 2 contained cells without Ad5, and Group 3 contained both cells and Ad5. The results showed nearly overlapping regression lines for Groups 1–3, whereas Group 4 exhibited a parallel but right-shifted curve with higher Ct values, indicative of PCR inhibition. This was unexpected, as Group 4 was theoretically the least prone to interference. A plausible explanation is that cellular components may neutralize the inhibitory substances present in the lysis buffer; their absence in Group 4 thus led to stronger inhibition and higher Ct values. We ultimately selected Group 3 for the standard curve preparation, as it shares identical background components (cells and Ad5) with the test wells, ensuring accurate rcAAV8 genome titer calculation. Repeated experiments confirmed the excellent reproducibility of this standard curve, with an LOQ of 38 copies, comparable to plasmid-based standards. Importantly, we observed that rcAAV samples intended for standard curves must be stored at high concentrations (RS1 level) and serially diluted immediately before use. The storage of pre-diluted series (RS1–RS8) led to a loss of qPCR linearity, with only the two highest concentrations yielding detectable amplification, indicating the instability of low-titer AAV samples during storage. Therefore, we strongly recommend storing samples at high titers and performing serial dilution only after thawing, immediately before adding the samples to cell plates.

For viral infection, parameters such as cell seeding density and infection duration were optimized. Infectious titers increased with higher cell numbers, likely because more cells provide greater opportunities for viral entry, thereby enhancing detection sensitivity. The simultaneous inoculation of cells and virus simplified the procedure without compromising performance. During the suspension stage, this approach promotes full contact between the virus and cells, increasing infection probability.

When applied to our rcAAV8 samples, the optimized method attained an intra-assay coefficient of variation (CV) below 40%. To further reduce intra-assay variability, rigorous sample mixing and careful pipetting during serial dilution and plate seeding are recommended. Additionally, including replicate plates within each experiment and averaging their results helped to mitigate variability and improve consistency across runs. Ultimately, when this method was used, an inter-assay CV as low as 11.4% was attained, which is markedly lower than the best-reported value (>30%) in the literature [24]. Although further optimization to reduce variability is warranted, the current result constitutes a substantial improvement on the conventional TCID_50_ assay. Since the assay targets the *rep2* sequence, it is equally applicable for determining the infectious titer of diverse rcAAV genotypes harboring this region, thereby providing a universal tool for calibrating recombinant rcAAV reference standard materials.

## 5. Conclusions

This study presents an optimized TCID_50_ assay for the accurate quantification of infectious rcAAV particles. Through the systematic refinement of key assay components, we have significantly improved the accuracy and reproducibility of this assay. The established protocol provides a critical technical foundation for developing qualified rcAAV reference standards, thereby enhancing the standardization and reliability of rcAAV testing. Moreover, this methodology offers a valuable reference for determining the infectious titer of other rAAV products, although the qPCR primer–probe sets must be tailored to the specific transgene sequence of interest, and further validation is required. Collectively, these advances substantially strengthen quality control measures for rAAV-based therapeutics.

## Figures and Tables

**Table 1:** Sequences of the primers and probes.

No.	Forward Primer (5′→3′)	Reverse Primer (5′→3′)	Probe (5′→3′)
*cap8*-1	5′-TCAGTTCCAGACCCTCAACC-3′	5′-GCCTTCGTTATTGTCTGCCA-3′	5′-VIC-CGCCACCGCCTGCAGCCATT-BHQ1-3′
*cap8*-2	5′-CGGACGTGTTCATGATTCCC-3′	5′-CCAGGCAGTAGAAGGAGGAG-3′	5′-VIC-CGTCCCACGGCCTGACTACCGT-BHQ1-3′
*cap8*-3	5′-GATCCTCCGACCACCTTCAA-3′	5′-TTCCTTCTGCAGCTCCCATT-3′	5′-VIC-CCACGCTGACCTGTCCGGTGCT-BHQ1-3′
*rep2*-1	5′-GAGAATTTACCGCGGGATCG-3′	5′-GTAGCACTCATCCACCACCT-3′	5′-FAM-CCCGCCTCCGGCGCCATTT C-BHQ1-3′
*rep2*-2	5′-AACTACCGGGAAGACCAACA-3′	5′-GTCGACACAGTCGTTGAAGG-3′	5′-FAM-TCGCGGAGGCCATAGCCCACA-BHQ1-3′
*rep2*-3	5′-CACACTGTGCCCTTCTAC-3′	5′-ACCAGATCACCATCTTGTC-3′	5′-FAM-AACTGGACCAATGAGAACTTTCC-BHQ1-3′
ITR	5′-GGAACCCTAGTGATGGAGTT-3′	5′-CGGCCTCAGTGAGCGA-3′	5′-FAM-CGAGCGCGCAGAGAGGGAGTG-BHQ1-3′

**Table 2:** Preparation of the standard curve from experimental groups.

Group No.	Day 1 Procedure	Day 3 Procedure
1	Cells^+^rcAAV^+^Ad5^−^: Co-seeded 50 μL of HEK293T cells (in 10% FBS-DMEM) and 50 μL of rcAAV dilutions (in Opti-MEM) into a 96-well plate.	Added 90 μL of lysis buffer → performed stepwise cell lysis → conducted qPCR analysis.
2	Cells^+^rcAAV^−^Ad5^−^: Co-seeded 50 μL of HEK293T cells (in 10% FBS-DMEM) and 50 μL of Opti-MEM into a 96-well plate.	Removed 50 μL of supernatant → added 50 μL of rcAAV dilutions (in Opti-MEM) → added 90 μL of lysis buffer → performed stepwise cell lysis → conducted qPCR analysis.
3	Cells^+^rcAAV^−^Ad5^+^: Co-seeded 50 μL of HEK293T cells (in 10% FBS-DMEM) and 50 μL of Ad5 (in Opti-MEM) into a 96-well plate.	Same as Group 2
4	Cells^−^rcAAV^−^Ad5^−^: Added 50 μL of 10% FBS-DMEM and 50 μL of Opti-MEM into a 96-well plate.	Same as Group 2

**Table 3:** Performance of standard curves from Group 3.

Test No.	Fitting Curve Equation (RS1~RS7)	R^2^	Amplification Efficiency (%)	Measured Value of RS6 (vg)	Measured Value of RS7 (vg)
1	y = −3.4212 logx + 41.66	0.9998	96.0	362.3	41.4
2	y = −3.4144 logx + 41.567	0.9998	96.3	357.4	41.7
3	y = −3.4652 logx + 41.061	0.9993	94.3	357.5	45.8
4	y = −3.4582 logx + 41.724	0.9992	94.6	442.1	38.3
5	y = −3.4592 logx + 41.203	0.9987	94.6	328.8	50.1
CV%	/	/	/	11.5	10.6

**Table 4:** Test results of the developed assay.

Test	Test Results (TCID_50_/mL)				Intra-Assay RSD (%)
	Plate 1	Plate 2	Plate 3	Mean	
1	5.22 × 10^8^	4.31 × 10^8^	6.32 × 10^8^	5.28 × 10^8^	19.1
2	4.31 × 10^8^	4.31 × 10^8^	7.66 × 10^8^	5.43 × 10^8^	35.7
3	4.31 × 10^8^	4.31 × 10^8^	6.32 × 10^8^	4.98 × 10^8^	23.4
4	7.66 × 10^8^	4.31 × 10^8^	4.31 × 10^8^	5.43 × 10^8^	35.7
5	4.31 × 10^8^	3.56 × 10^8^	4.31 × 10^8^	4.06 × 10^8^	10.7
Mean (TCID_50_/mL)				5.04 × 10^8^	/
Inter-assay RSD (%)				11.4	/

## Data Availability

The original contributions presented in this study are included in the article. Further inquiries can be directed to the corresponding authors.

## Abbreviations

The following abbreviations are used in this manuscript:

TCID_50_	Tissue culture infection dose 50%
AAV	Adeno-associated virus
rcAAV	Replication-competent adeno-associated virus
LOQ	Limit of quantitation
